# The Na_v_1.9 channel regulates colonic motility in mice

**DOI:** 10.3389/fnins.2013.00058

**Published:** 2013-04-15

**Authors:** Carine Copel, Nadine Clerc, Nancy Osorio, Patrick Delmas, Bruno Mazet

**Affiliations:** Aix Marseille Université, CNRS, CRN2M UMR 7286Marseille, France

**Keywords:** Na_v_1.9 channel, migrating motor complex, colon, enteric, neuron

## Abstract

The colonic migrating motor complex (CMMC) is a major pattern of motility that is entirely generated and organized by the enteric nervous system. We have previously demonstrated that the Na_v_1.9 channel underlies a tetrodotoxin-resistant sodium current which modulates the excitability of enteric neurons. The aim of this study was to observe the effect of loss of the Na_v_1.9 channel in enteric neurons on mouse colonic motility *in vitro*. The mechanical activity of the circular muscle was simultaneously recorded from three sites, namely, proximal, mid- and distal, along the whole colon of male, age-matched wild-type and Na_v_1.9 null mice. Spontaneous CMMCs were observed in all preparations. The mean frequency of CMMCs was significantly higher in the Na_v_1.9 null mice (one every 2.87 ± 0.1 min compared to one every 3.96 ± 0.23 min in the wild type). The mean duration of CMMCs was shorter and the mean area-under-contraction was larger in the Na_v_1.9 null mice compared to the wild type. In addition, CMMCs propagated preferentially in an aboral direction in the Na_v_1.9 null mice. Our study demonstrates that CMMCs do occur in mice lacking the Na_v_1.9 channel, but their characteristics are significantly different from controls. Up to now, the Na_v_1.9 channel was mainly associated with nociceptive neurons and involved in their hyperexcitability after inflammation. Our result shows for the first time a role for the Na_v_1.9 channel in a complex colonic motor pattern.

## Introduction

Sodium (Na^+^) channels are essential for action potential generation and propagation in nerve cells. Different Na^+^ channel subunits (Na_v_), among which is Na_v_1.9, are expressed in sensory neurons of dorsal root (DRG) and trigeminal ganglia (Dib-Hajj et al., 2002; Cummins et al., 2007). Na_v_1.9 is predominantly associated with nociceptive neurons and thus may play a crucial role in pain (Dib-Hajj et al., 2002; Rogers et al., 2006; Cummins et al., 2007). However, Na_v_1.9 may also play a role in normal conditions since it is present in both non-nociceptive and nociceptive lung-specific nodose ganglion neurons (Kwong et al., 2008). Na_v_1.9 supports a tetrodotoxin-resistant (TTXr) Na^+^ current (Priest et al., 2005; Amaya et al., 2006; Maingret et al., 2008). Because of its slow activation kinetics, this TTXr Na^+^ current is probably not substantially involved in the generation of action potentials. Rather, it may set the resting membrane potential, determine the shape of the action potential and tune the voltage threshold for the firing of action potentials (Dib-Hajj et al., 2002; Coste et al., 2004; Rogers et al., 2006; Cummins et al., 2007; Maingret et al., 2008). More specifically, it has been shown that Na_v_1.9 contributes to the hyperexcitability of DRG neurons in inflammatory conditions (Amaya et al., 2006; Cummins et al., 2007; Maingret et al., 2008; Liu and Wood, 2011). However, so far, the very few functional studies related to gastrointestinal (GI) functions in the mouse conclude that the inflammatory (colon; Beyak et al., 2004) or post-inflammatory (small intestine; Hillsley et al., 2006) hyperexcitability of DRG neurons supplying the GI wall does not involve Na_v_1.9. Recently, it was shown that Na_v_1.9 is also expressed in neurons of the enteric nervous system (ENS) lying in the GI tract (Rugiero et al., 2003; Padilla et al., 2007). The ENS consists of intrinsic neural circuitry that arises from two major plexuses and is responsible for the generation of complex motor patterns as well as the control of fluid secretion (Schemann and Neunlist, 2004; Furness, 2006). Interestingly, the presence of Na_v_1.9 is restricted to Dogiel type II/AH as typical sensory neurons in the myenteric and submucosal enteric plexuses (Rugiero et al., 2003; Padilla et al., 2007). Moreover, Na_v_1.9 modulates the excitability of myenteric sensory neurons and because the TTXr current is strongly amplified by the activation of NK3 receptors, it has been suggested to facilitate slow synaptic transmission within the myenteric network (Rugiero et al., 2003; Copel et al., 2009). Any change in excitability of enteric sensory neurons may thus shape their integrative properties and ultimately affect gut motility and secretion.

The colonic migrating motor complex (CMMC) is a major pattern of spontaneous contractile activity that consists of a cyclical contraction that can propagate over significant lengths of the large intestine (Spencer, 2001). CMMCs are well-defined in the mouse colon *in vitro* (Fida et al., 1997, 2000; Bush et al., 2000, 2001; Brierley et al., 2001; Powell and Bywater, 2001; Spencer, 2001; Powell et al., 2002, 2003; Spencer and Bywater, 2002) and *in vivo* (Gourcerol et al., 2009) and occur during both the fasted and fed state. This motor pattern is entirely generated and organized by the ENS (Spencer, 2001; Furness, 2006) and drives digestive content propulsion (Spencer, 2001; Furness, 2006; Heredia et al., 2009). Although the enteric mechanisms underlying the CMMC are unclear, it is initiated through the activation of myenteric neurons (Spencer et al., 2005; Heredia et al., 2009; Bayguinov et al., 2010; Dickson et al., 2010b; Keating and Spencer, 2010).

In light of the fact that Na_v_1.9 plays an important role in the control of excitability of myenteric sensory neurons, the aim of the present study was to characterize if genetic deletion of Na_v_1.9 altered the characteristics of CMMCs in isolated mouse colon. We have used tension recordings from the circular muscle of the full length isolated colon from wild-type and Na_v_1.9 null mice.

## Materials and methods

All procedures were in accordance with the directives of the French Ministry of Agriculture and Fisheries and the European Community Council (86/609/EEC).

### Animals

Male, age-matched, non-fasted C57Bl/6 (wild type: +/+) and Na_v_1.9 null (−/−) mice were used. Briefly, heterozygous mice generated by Glaxo Smith Kline were crossed with wild-type C57Bl/6 mice (Na_v_1.9^+/+^) to generate the homozygous Na_v_1.9^−/−^ mice employed in this study (see Amaya et al., 2006 for details).

### Tissue preparation

#### Mechanical recording experiments

Mice were killed by cervical dislocation. The entire GI tract was quickly excised and placed in a Sylgard-based organ bath containing a Kreb's solution (KS; see composition below) at 4°C and equilibrated with 95% O_2_-5% CO_2_. The whole colon (≈6–7 cm) was dissected off and the luminal content was gently flushed using a KS-filled syringe. A stainless steel, side-hole tube (length: 60 mm; external diameter: 2.34 mm; wall thickness: 0.32 mm) was inserted through the length of the colon. The male end of a Luer Lock was inserted into each end of the tube and the colon was ligated on with cotton thread. Three stainless steel hooks made from minute pins (diameter: 0.15 mm) were inserted through the muscularis externa at the mesenteric border. The first one was placed precisely at the mid-length of the colon and the other two were placed 1.5 cm apart in the oral and anal direction, respectively. The colon was then placed in a recording chamber continuously perfused (3 mL/min) with gassed KS. The Luer Lock at each end was connected to a glass cannula, the anal one being equipped with a pressure transducer to adjust and monitor the intraluminal pressure of the organ. The pressure was set to 1 cm H_2_O for all preparations. Each hook was connected via a loop made of stainless steel wire (diameter: 0.15 mm) to a Grass FT03 transducer (Grass Instruments, Quincy, MA, USA). The initial tension at the three sites of recording of mechanical activity of the circular muscle was set to 500 mg. The KS in the bath was progressively warmed up to 36 ± 0.1°C. The tension, pressure and temperature were then continuously recorded and later analysis was performed on a PC running AcqKnowledge software 3.7.3 (Biopac Systems, Goleta, CA, USA). The tissue was allowed to equilibrate until a steady spontaneous CMMC activity appeared.

#### Immunohistochemistry

The colon was prepared as above except that 1 μM atropine and 3 μM nicardipine were added in KS to prevent muscle contraction. The organ was opened along the mesenteric border, stretched and pinned mucosal side up in a Sylgard-based Petri dish containing KS. After removing the mucosa, the tissue was unpinned, turned over and pinned again. It was rinsed in phosphate-buffered saline (PBS; 0.9% NaCl in 0.01 M sodium phosphate buffer, pH 7.2), cryoprotected by immersion for 3 h into PBS containing successively 20 and 40 % sucrose. The tissue was then mounted with longitudinal muscle up on Superfrost slides (D. Dutscher SAS, France) and frozen. In the rat and the guinea pig, the expression of Na_v_1.9 is restricted to sensory neurons (Lancaster and Weinreich, 2001; Dib-Hajj et al., 2002; Rugiero et al., 2003; Li and Schild, 2007; Padilla et al., 2007; Kwong et al., 2008). Enteric sensory neurons have a distinctive shape, with large round or oval cell bodies and multiple axons, that can be visualized in different species, although not selectively, using anti-neurofilament antibodies (Brehmer et al., 2002, 2004; Padilla et al., 2007; Wolf et al., 2007). Immunodetection of both Na_v_1.9 and NF-200 proteins was thus performed using an anti-Na_v_1.9 antibody raised in rabbit against the amino acid sequence _865_KDSILPDARPWKEYD_879_ located in the intracellular II-III linker of the rat Na_v_1.9 alpha subunit (1/100; anti-Na_v_1.9 as L-23; Padilla et al., 2007) and a monoclonal anti-NF-200 antibody raised in mouse (1/400; Sigma). Before exposure to primary antibodies, the mounted tissues were preincubated for 1 h in PBS containing 3% bovine serum albumin and 0.1% Triton ×-100 (blocking buffer) in order to reduce non-specific binding. Then, they were exposed overnight at 4°C to the primary antibodies diluted in the blocking buffer. Goat anti-rabbit IgG-FITC (1/200) and goat anti-mouse IgG-TRITC (1/400), both from Jackson ImmunoResearch (West Grove, PA, USA), were used as secondary antibodies. After washing the tissues several times in PBS, the secondary antibodies were diluted in the blocking buffer and applied to the tissues for 1 h at room temperature. Finally, the tissues were mounted in PBS-50% glycerol. Confocal image acquisition was performed on a Leica TCS SP2 laser scanning microscope (Leica Microsystems, Mannheim, Germany) using a 488 nm band of an Ar laser for excitation of FITC and a 568 nm band of an He-Ne laser for excitation of TRITC. Images were acquired by sequential scanning using a ×40 (numerical aperture = 1.25) or ×63 (numerical aperture = 1.32) oil immersion objective and processed with Adobe Photoshop.

### Solutions and drugs

The composition of the KS was (in mM): NaCl 120; KCl, 5; NaH_2_PO_4_, 1; MgSO_4_, 1; CaCl_2_, 2.5; NaHCO_3_, 25; glucose, 11. The solution was gassed continuously with 95% O_2_-5% CO_2_ to give a final pH of 7.3–7.4. The salts were all from Sigma (St. Louis, MO, USA). Tetrodotoxin (TTX) was from Alomone Laboratories (Jerusalem, Israël).

### Analysis of data and statistics

In many preparations, within a few tens of minutes, an intraluminal blockage due to the accumulation of remnants of digestive contents and mucosal secretion obstructed the anal output. In these cases, the intestinal lumen needed to be flushed to free the lumen thus disturbing the progress of the spontaneous CMMC activity. Data from these preparations were not included in this study. Cyclical, long-duration contractions which alternated with periods of relative quiescence and which were observed to occur at more than one recording site were considered to be CMMCs (Fida et al., 1997). The period, duration, area, and maximum amplitude of CMMCs were measured for all recording sites. To perform measurements, the starting and ending points of CMMCs were determined from the average baseline tension during the relative quiescence between CMMCs. The period between CMMCs was determined by the time between the onset of rising phases (starting points) of consecutive contractions. The duration and area-under-contraction were determined between the starting point and the end of the falling phase (ending point) of the CMMC. The maximum amplitude was determined between the starting point and the peak of the CMMC. The recording site where the CMMC occurred first determined the site of origin. CMMCs were considered to occur simultaneously when no delay was detectable between sites. The propagation velocity was determined by dividing the distance between the recording sites (1.5 cm) over the time from the starting point of the CMMC at one recording site to the same point at another recording site. Because some of these parameters usually wane with time (see Bush et al., 2001) and in order to allow accurate comparisons between types of mice, in both groups analysis started at the same time-point from the beginning of the recording session and lasted for 20–50 min. Therefore, only preparations from which time-matched data were obtained were included in this study. Data are expressed as mean ± standard error of the mean (SEM). “n” refers to the number of preparations. Statistical analysis was performed using GraphPad Instat 3.06 (GraphPad Software, San Diego, CA, USA). Student's two-tailed unpaired *t* tests or multiple comparison tests were used where appropriate. The site of origin of CMMCs was tested for significant changes between the two types of mice using a Fisher Exact test. *P* < 0.05 was considered significant.

## Results

### Immunohistochemistry

**Figure 1:**
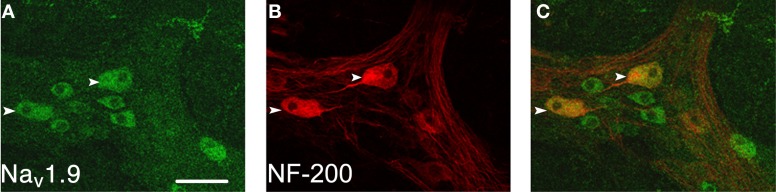
**Localization of Na_v_1.9 immunoreactivity in the myenteric plexus of the mouse colon.** Na_v_1.9-like immunoreactivity, detected with L-23 AB **(A)**, is located in large neurons (arrowheads) that are NF-200 positive **(B)** and in smaller NF-200 negative neurons. These two neuron populations are well evidenced on the overlay **(C)**. Scale bar = 50 μm.

Na_v_1.9 immunoreactivity was present within the myenteric plexus of the colon of +/+ mice (*n* = 6; Figure 1). It was diffuse within some nerve cell bodies with no extension within processes. It was found that 31.5% of myenteric neurons that were immunoreactive for Na_v_1.9 were also immunoreactive to NF-200. The NF-200-positive neurons had large and smooth cell bodies indicative of Dogiel type II neurons. For these neurons, it was possible to accurately measure cell body sizes; the major axis was 34.7 ± 6.4 μm and the minor axis was 16.9 ± 3.8 μm (for 28 neurons from 6 preparations). The other neurons appear smaller and their type remains undetermined. No staining was detected in tissues incubated with the secondary antibodies alone.

### Spontaneous mechanical activity

#### General observations

**Figure 2:**
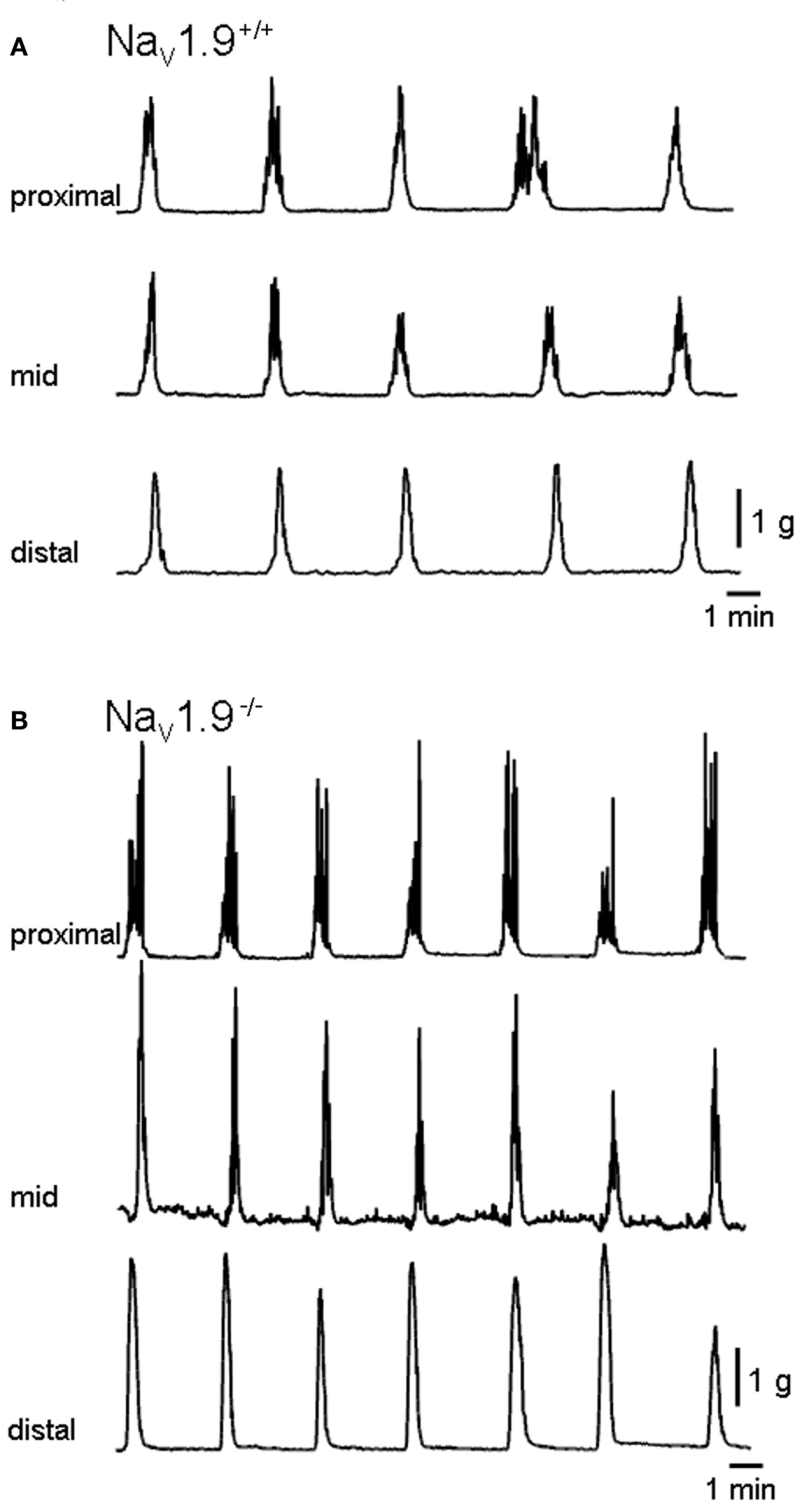
**Mechanical recordings made simultaneously from three sites of the colon. (A)** and **(B)** are typical traces of migrating motor complexes in the colon (CMMCs) of wild (+/+) and Na_v_1.9^−/−^ mice, respectively. In Na_v_1.9^−/−^ mice, the interval between CMMCs and their duration were shorter and the area higher than in wild-type mice.

Spontaneous CMMCs were recorded from the colon of all preparations (Figure 2). CMMCs in the proximal and, most of the time, mid-colon displayed phasic contractions superimposed on a slower contraction. Phasic contractions were infrequently observed in the distal colon. CMMCs were abolished in the presence of TTX (1 μM) in both +/+ and Na_v_1.9^−/−^ mice (*n* = 3 for each; data not shown).

The length of the colon was not significantly different between the +/+ (6.71 ± 0.24 cm) and the Na_v_1.9^−/−^ mice (6.65 ± 0.3 cm) (*P* > 0.05).

#### Mechanical activity of the colon of +/+ mice

**Figure 3:**
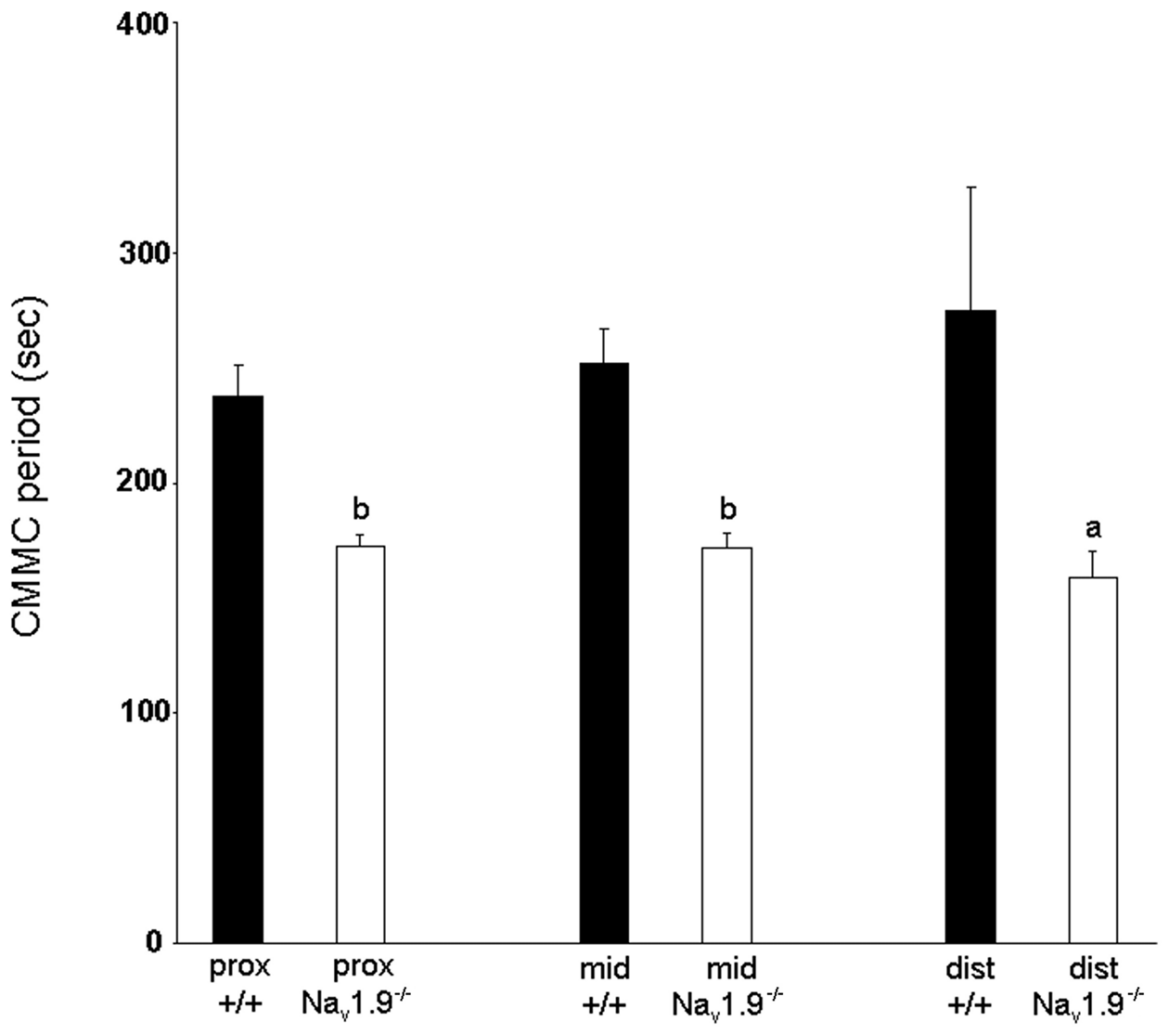
**Graphic representation of the period of CMMCs in the proximal, mid- and distal colon of wild-type and Na_v_1.9^−/−^ mice.** The period of CMMC in Na_v_1.9^−/−^ mice was found to be significantly shorter than in +/+ mice. ^a^*P* < 0.05, ^b^*P* < 0.01 vs. +/+ mice.

The mean period between CMMCs was 237.6 ± 13.7 s in the proximal colon, 251.9 ± 15.4 s in the mid-colon and 274.8 ± 53.3 s in the distal colon (Figure 3). No significant difference was observed in the period of CMMCs between regions (*n* = 12; *P* > 0.05).

**Figure 4:**
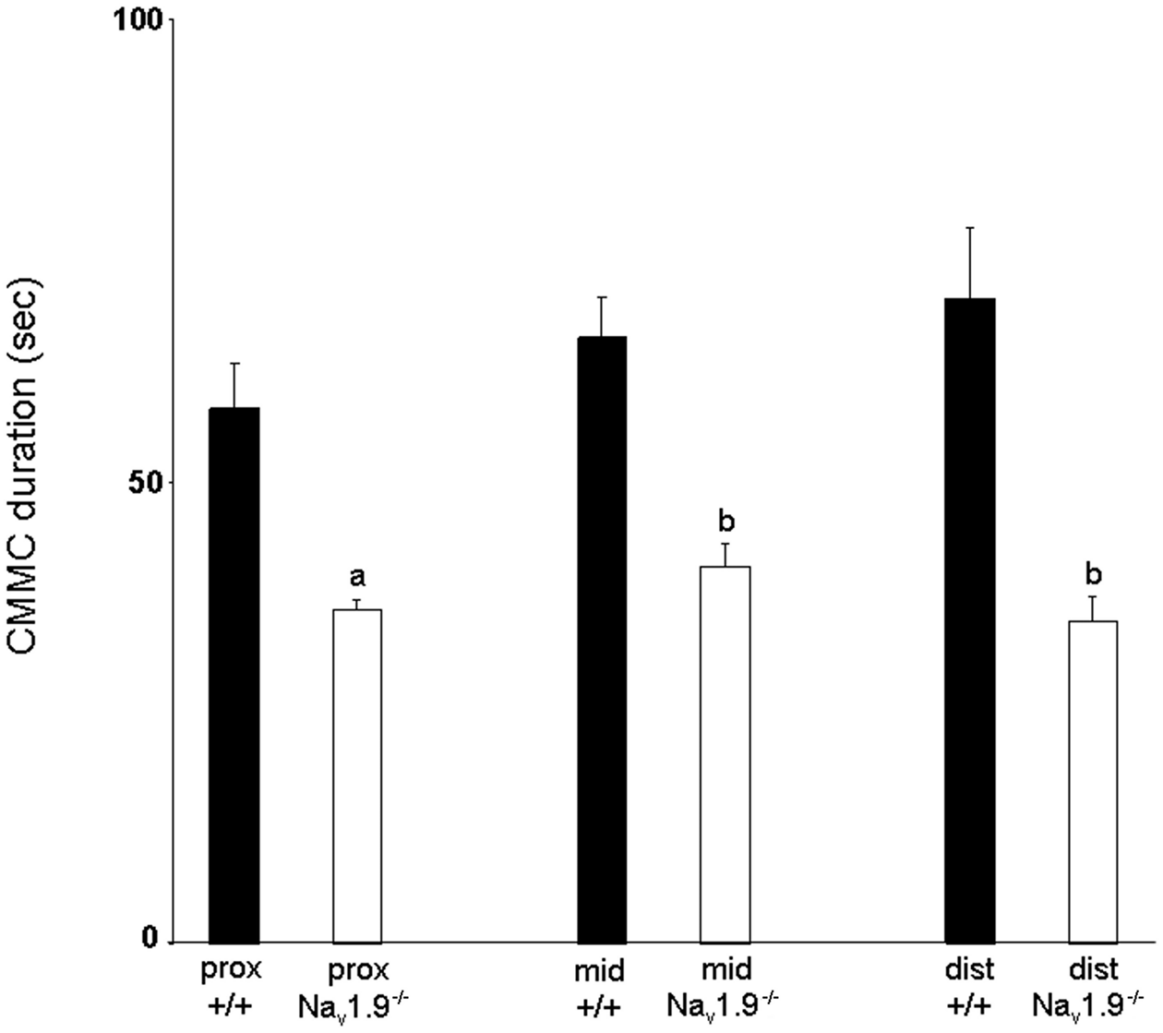
**Graphic representation of the duration of CMMCs in the proximal, mid- and distal colon of wild-type and Na_v_1.9^−/−^ mice.** The duration of CMMCs in Na_v_1.9^−/−^ mice was found to be significantly shorter than in +/+ mice. ^a^*P* < 0.05, ^b^*P* < 0.01 vs. +/+ mice.

The mean duration of CMMCs in the proximal colon was 57.9 ± 4.9 s, 65.6 ± 4.3 s in the mid- and 69.8 ± 7.6 s in the distal colon (Figure 4). There was no significant difference in the duration of CMMCs between regions (*P* > 0.05).

**Figure 5:**
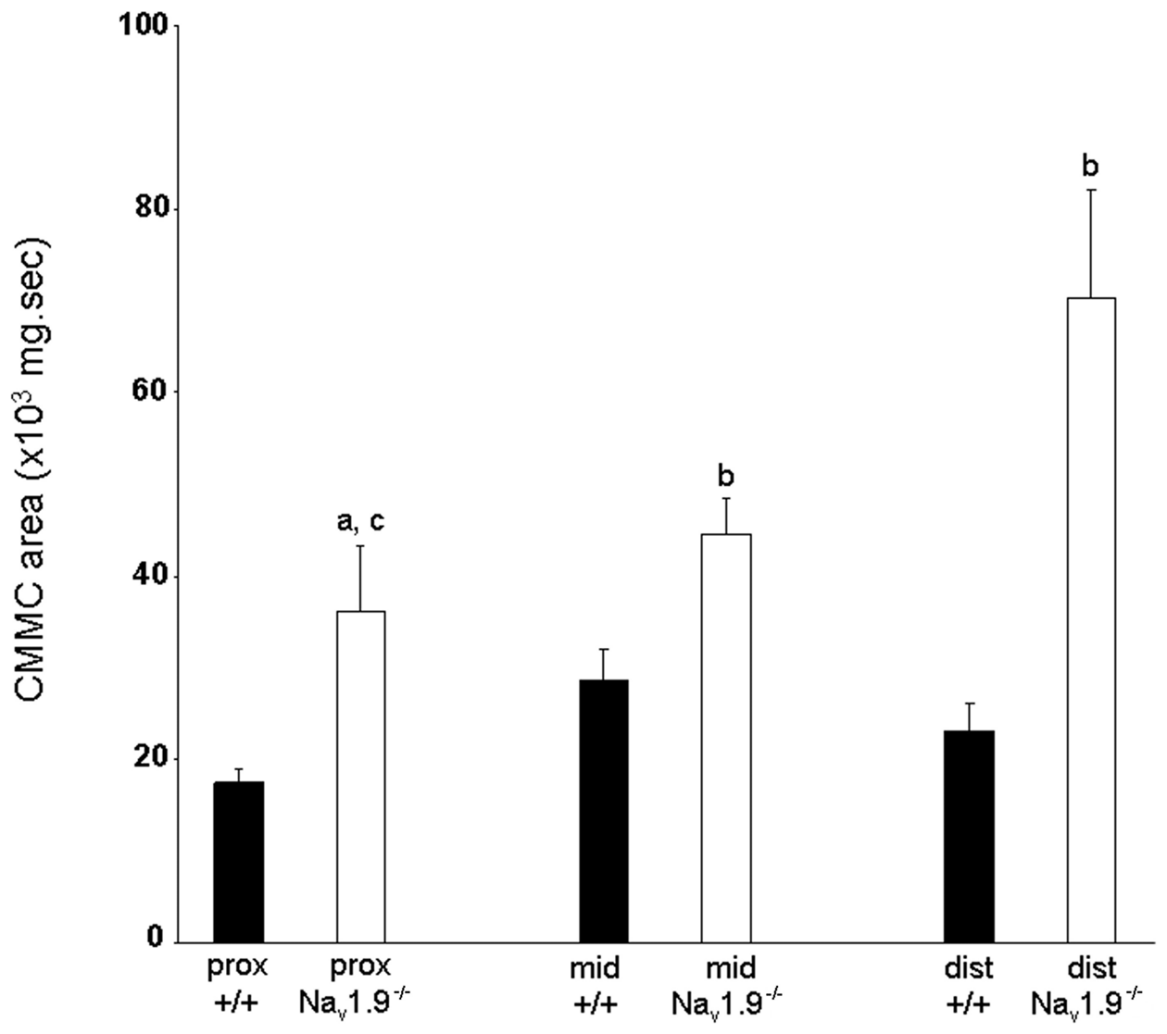
**Graphic representation of the area of CMMCs in the proximal, mid- and distal colon of wild-type and Na_v_1.9^−/−^ mice.** The area of CMMCs in Na_v_1.9^−/−^ mice was found to be significantly larger than in +/+ mice. In Na_v_1.9^−/−^ mice, the area of CMMCs in the proximal colon was significantly smaller than in the distal region. ^a^*P* < 0.05, ^b^*P* < 0.01 vs. +/+ mice; ^c^*P* < 0.01 proximal vs. distal colon in Na_v_1.9^−/−^ mice.

The mean area of CMMCs in the proximal colon was 17349.9 ± 1521 mg.sec, 28674 ± 3344 mg.sec in the mid- and 23065 ± 3005 mg.sec in the distal colon in Na_v_1.9^+/+^ mice (Figure 5). The area of CMMCs did not vary significantly along the length of the colon (*P* > 0.05). There was also no significant difference in the maximum amplitude of CMMCs between regions (proximal: 2.37 ± 0.22 g; mid: 2.26 ± 0.18 g; distal: 2.19 ± 0.32 g) (*P* > 0.05).

#### Mechanical activity of the colon of Na_v_1.9^−/−^ mice

The mean period between CMMCs was 172 ± 5.6 s in the proximal colon, 171.9 ± 6 s in the mid- and 158.6 ± 11.4 s in the distal colon (Figure 3). There was no significant difference in the period of CMMCs between regions (*n* = 9; *P* > 0.05). However, the mean period between CMMCs was significantly shorter in Na_v_1.9^−/−^ tissues than in +/+ mice for all regions (*P* < 0.05).

The mean duration of CMMCs was 36.1 ± 1.3 s in the proximal colon, 40.9 ± 2.6 s in the mid- and 34.8 ± 2.6 s in the distal colon (Figure 4). No significant difference was observed in the duration of CMMCs between regions (*P* > 0.05) but we found that the duration of CMMCs was significantly shorter in every region in Na_v_1.9^−/−^ mice when compared to the corresponding region in +/+ tissues (*P* < 0.05).

The mean area of CMMCs was 35977 ± 7366 mg.sec in the proximal colon, 44501 ± 3983 mg.sec in the mid- and 70320 ± 11761 mg.sec in the distal colon (Figure 5). The area of CMMCs in the proximal colon was significantly smaller than in the distal colon (*P* < 0.05). We also found that the area of CMMCs was significantly larger in every region in Na_v_1.9^−/−^ colons when compared to the corresponding regions from +/+ mice (*P* < 0.05).

#### Site of origin and velocity

**Figure 6:**
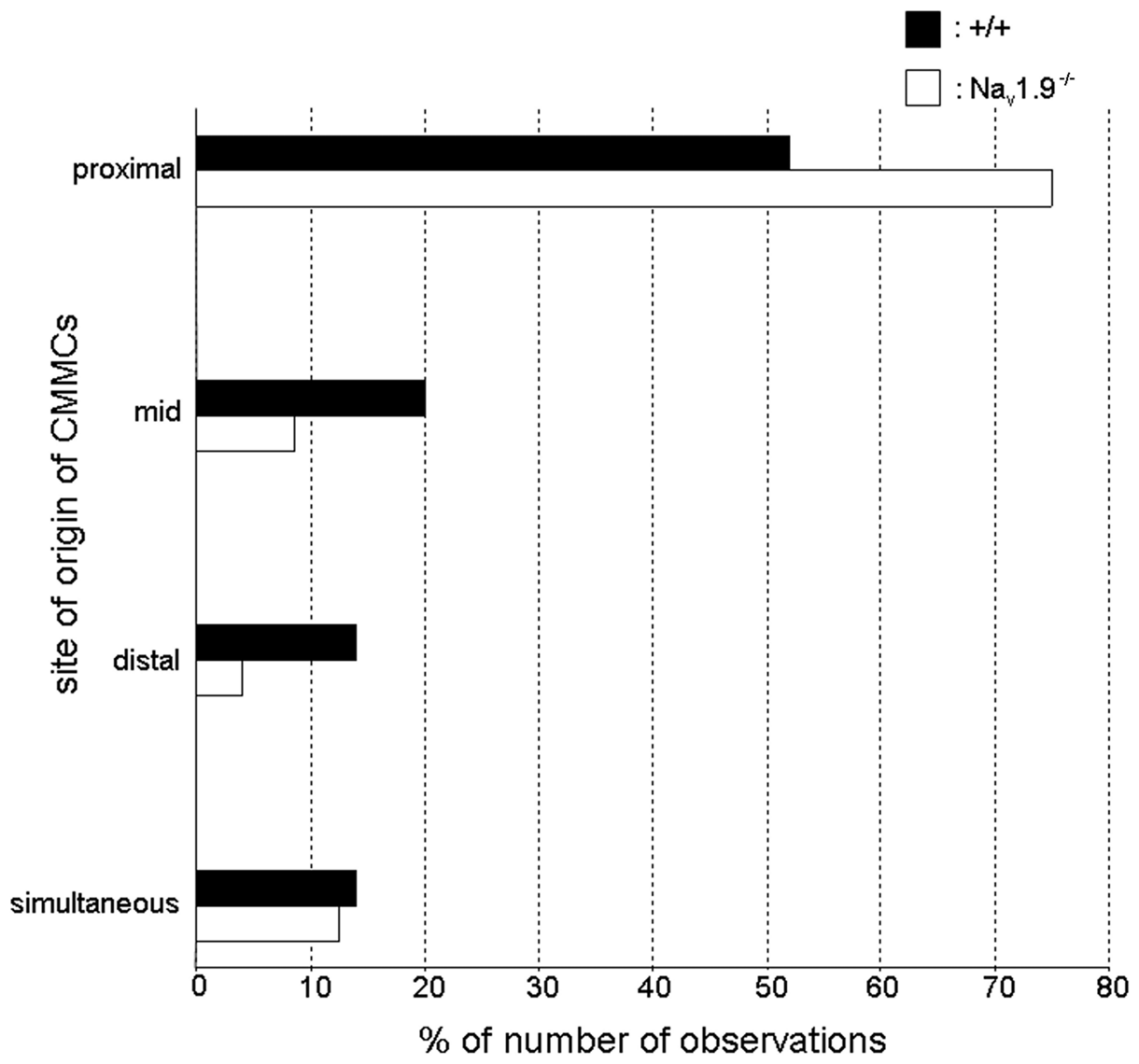
**Graphic representation of the site of origin of CMMCs in wild-type and Na_v_1.9^−/−^ mice.** In +/+ mice, about 50% of CMMCs propagated from the proximal region. This proportion increased to 75% in Na_v_1.9^−/−^ mice. The site of origin of CMMCs was significantly different in the colon of Na_v_1.9^−/−^ mice compared to that of +/+ mice (*P* < 0.05).

In both +/+ and Na_v_1.9^−/−^ mice, the site of origin of CMMCs varied between preparations and during the recording period in a same preparation (Figure 6).

The propagation was predominantly from the oral to the more anal regions of the colon in both types of mice but there was a shift of the site of origin to the proximal colon in Na_v_1.9^−/−^ mice (75%; 54 out of 72 CMMCs) compared to +/+ mice (52%; 56 out of 108 CMMCs). In addition, in +/+ mice, 41% of these CMMCs (23 out of 56) did not migrate up to the distal colon and this proportion decreased to 16.7% (9 out of 54 CMMCs) in Na_v_1.9^−/−^ mice. In the other cases, the site of origin of CMMCs was the mid-colon [+/+: 20% (22 out of 108 CMMCs, among which 59% migrated only in the oral direction; Na_v_1.9^−/−^: 8.5% (6 out of 54 CMMCs)] or the distal colon [+/+: 14% (15 out of 108 CMMCs); Na_v_1.9^−/−^: 4% (3 out of 72 CMMCs)]. Also, CMMCs sometimes occurred simultaneously in two or three regions of the colon (+/+: 14% (15 out of 108 CMMCs); Na_v_1.9^−/−^: 12.5% (9 out of 72 CMMCs)]. The changes in the site of origin in Na_v_1.9^−/−^ mice were statistically significant when compared to +/+ mice (*P* < 0.05).

In +/+ mice, the mean propagation velocity of anally migrating CMMCs was 1.05 ± 0.18 mm/s from the proximal to mid-colon and 0.87 ± 0.1 mm/s from the mid- to distal colon. The mean velocity was not significantly different between these two paths (*P* > 0.05). For anally migrating CMMCs in Na_v_1.9^−/−^mice, the mean velocity from the proximal to mid-colon (1.33 ± 0.19 mm/s) was also not significantly different from that of the mid- to distal colon (1.76 ± 0.34 mm/s) (*P* > 0.05). There was no significant difference in mean velocities between +/+ and Na_v_1.9^−/−^ mice (*P* > 0.05) during anally migrating CMMCs. The mean propagation velocity of orally migrating CMMCs in +/+ mice was not significantly different between the distal to mid-colon (2.28 ± 0.69 mm/s) and the mid- to proximal colon (2.66 ± 0.46 mm/s) (*P* > 0.05). However, these velocities were significantly faster than those of the corresponding paths during anally migrating CMMCs (*P* < 0.05). In Na_v_1.9^−/−^ mice, the mean propagation velocity from the distal to mid-colon (2.06 ± 0.17 mm/s) was apparently faster than from the mid- to proximal colon (0.96 ± 0.23 mm/s) and for both paths, the mean velocity was not significantly different from the corresponding contractions during anally migrating CMMCs. However, the number of orally migrating CMMCs in Na_v_1.9^−/−^ mice was too low (see above) to test for statistical significance.

## Discussion

The present study shows that *in vitro* CMMCs are present in mice lacking Na_v_1.9, however there are some major differences in their characteristics when compared to control mice. This result assigns a role to Na_v_1.9 in basal functional conditions for the first time.

Our data first reveal the presence of Na_v_1.9 in myenteric neurons of the mouse colon. Many Na_v_1.9-immunoreactive neurons have clearly the morphology of Dogiel type II neurons that have been identified as intrinsic primary afferent neurons in other species (Furness et al., 2004; Furness, 2006). On the basis of both morphological and electrophysiological data, these neurons are assumed to be sensory neurons in the mouse colon as well (Furness et al., 2004; Nurgali et al., 2004). Na_v_1.9 has been strictly localized in sensory neurons in every system so far studied, including the ENS (see Material and Methods; Rugiero et al., 2003; Padilla et al., 2007); however, we cannot positively exclude that interneurons and/or motor neurons (some of which having a sensory function; Spencer and Smith, 2004; Smith et al., 2007; Mazzuoli and Schemann, 2012) also express Na_v_1.9 in the mouse colon. As already stated, Na_v_1.9 does not support the hyperexcitability of extrinsic sensory (DRG) neurons supplying the GI wall in inflammatory models (Beyak et al., 2004; Hillsley et al., 2006). Thus, these conditions come together to envisage a role for Na_v_1.9 in normal gut functions. In Na_v_1.9^−/−^ mice which lack TTXr Na^+^ current (Amaya et al., 2006; Maingret et al., 2008), CMMCs occur at a higher frequency and are more forcible compared to +/+ mice. In addition, the main site of origin of CMMCs clearly changes to the oral end of the organ.

Although still unclear, the mechanisms supporting the initiation, generation, and propagation of the CMMC have recently received much attention (Spencer, 2001; Smith et al., 2007). The neural circuitry required for generating the CMMC is within the myenteric plexus and/or muscularis externa and myenteric neurons as sensory neurons (Heredia et al., 2009; Bayguinov et al., 2010; Dickson et al., 2010b) and interneurons (Spencer et al., 2005) are considered to play a key role.

Changes in many parameters of the CMMC have already been reported in various conditions and in response to pharmacological agents or to mechanical stimulation. Although no direct evidence was provided, the release of serotonin (5-HT) from enterochromaffin cells (EC) in the mucosa has been suggested to trigger spontaneous CMMCs (Heredia et al., 2009; Dickson et al., 2010b). In contrast, other studies indicate that the mucosa is not necessary for the generation of spontaneous CMMCs because they still occur after it is removed, and all 5-HT release has ceased; however, removal of the mucosa (involving 5-HT or another substance) modulates the frequency of CMMCs (Keating and Spencer, 2010; Zagorodnyuk and Spencer, 2011). Because Na_v_1.9 is found in myenteric neurons and has not been described in EC (see Padilla et al., 2007), we can exclude that the observed effects on the frequency of CMMCs in Na_v_1.9^−/−^ mice involve a modulation of the release of a substance from EC. At present, there is an overall agreement that spontaneous CMMCs are indeed generated by the activation of myenteric neurons (Heredia et al., 2009; Bayguinov et al., 2010; Dickson et al., 2010b; Keating and Spencer, 2010) and requires removal of a tonic inhibition of the muscle (disinhibition) (Lyster et al., 1995; Fida et al., 1997; Spencer et al., 1998; Powell and Bywater, 2001; Bayguinov et al., 2010; Dickson et al., 2010b). The triggering output from the myenteric sensory neurons activates, by synchronizing the activity of interneurons, both ascending excitatory and descending inhibitory pathways, in addition to disinhibition (Spencer et al., 2005; Heredia et al., 2009; Bayguinov et al., 2010; Dickson et al., 2010a,b). Moreover, activation of local intrinsic reflexes has significant impact on the progress of the CMMC and can even generate CMMCs under particular circumstances (Heredia et al., 2009). Despite the high degree of complexity in the enteric circuitry with regard to neuronal interconnections and the variety of neurotransmitters (Furness, 2006; Bayguinov et al., 2010; Dickson et al., 2010b), some points can be worthily discussed on the basis of the available literature.

The role of enteric neurons, specifically myenteric AH and interneurons, in initiating spontaneous CMMCs has received specific attention. We can first consider the possibility that myenteric AH neurons are involved in the generation and propagation of the CMMC (Smith et al., 2007; Heredia et al., 2009; Bayguinov et al., 2010). These neurons are interconnected and synaptic transmission between them largely involves slow EPSP (Kunze et al., 1993; Bertrand et al., 1997, 2000; Hillsley et al., 2000; Monro et al., 2005) that would thus be essential for the generation of the CMMC (Heredia et al., 2009; Bayguinov et al., 2010; Dickson et al., 2010b). Its unique electrophysiological properties (Cummins et al., 1999; Dib-Hajj et al., 2002; Rugiero et al., 2002, 2003; Coste et al., 2004; Maingret et al., 2008) allow Na_v_1.9 to sustain a persistent, TTXr Na^+^ current at rest. In other words, at resting membrane potential, the Na_v_1.9 current can maintain neurons in a depolarized state. Consequently, as the first and probably simplest step, it is necessary to consider the potential impact of the loss of Na_v_1.9 on the activity of AH neurons and their ability to transmit information. It may appear counterintuitive to observe an increase in CMMC activity while a key component supporting neuronal excitability as Na_v_1.9 is lacking. At least two hypotheses can be considered. Firstly, it has been suggested that the loss of Na_v_1.9 could produce an hyperpolarization (Herzog et al., 2001; Baker et al., 2003; Rush et al., 2007) that would remove inactivation on TTX-sensitive Na^+^ channels thus increasing neuronal excitability (Dib-Hajj et al., 2002). Consequently, the excitability of myenteric neurons equipped with Na_v_1.9 might be increased in Na_v_1.9^−/−^ mice and explain at least the increase of CMMC frequency. A second alternative leads to consider that the excitability of neurons lacking Na_v_1.9 would be actually decreased (Maingret et al., 2008). We can consider the possibility that in addition to the “classical” AH sensory neurons, interneurons are endowed with Na_v_1.9. Interestingly, recent cumulative evidence indicates that interneurons may have sensory functions (Spencer and Smith, 2004; Dickson et al., 2007; Smith et al., 2007; Mazzuoli and Schemann, 2012) and are major players involved in the generation of the CMMC (Spencer et al., 2005; Bayguinov et al., 2010; Dickson et al., 2010b; Keating and Spencer, 2010). At this point, it is necessary to consider recent hypotheses that have emerged mainly from pharmacological studies. 5-HT-containing interneurons expressing intrinsic, ongoing activity (Dickson et al., 2010b) can be hypothesized to drive nitric oxide synthase (NOS)-containing inhibitory motor neurons responsible for the tonic inhibition of the muscle between CMMCs (Bywater et al., 1989; Lyster et al., 1995; Spencer et al., 1998; Bayguinov et al., 2010; Dickson et al., 2010a) although a concern with this hypothesis is that despite the presence of 5-HT-containing neurons in the mouse colon, no synaptic potential mediated by endogenous 5-HT has ever been recorded in mouse enteric neurons (Furukawa et al., 1986; Nurgali et al., 2004). 5-HT can depolarize, but very interestingly also hyperpolarize and inhibit neurotransmitter release from AH sensory neurons depending on the subtypes of 5-HT receptors involved (see Dickson et al., 2010b). Whether only one or both categories among these inter- or motor neurons are equipped with Na_v_1.9, a decrease in their excitability resulting from the loss of Na_v_1.9 would undoubtedly weaken the tonic, nitrergic inhibition that is normally removed to allow the CMMC to develop and facilitate the output from AH sensory neurons. The increase in activity of AH neurons together with a “disinhibited” colon would favor excitatory pathways and therefore the development and strength of the CMMC as observed in Na_v_1.9^−/−^ mice. This view is further corroborated by the fact that non spontaneous CMMCs are readily evoked by mechanical stimulation in the distal colon because of a large mobilization of ascending excitatory pathways (Bayguinov et al., 2010). Indeed, spontaneous CMMCs in the colon of Na_v_1.9^−/−^ mice arise preferentially in the proximal colon as if a more prominent ascending excitation from distal parts took place. Also, the loss of Na_v_1.9 in interneurons could affect the synchronization of their firing thus compromising the generation of the CMMC (Spencer et al., 2005). To investigate the mechanisms supporting the changes in CMMCs observed in Na_v_1.9^−/−^ mice, it will be necessary to directly evaluate the excitability of enteric neurons lacking Na_v_1.9. In addition, because it is a major transmitter involved in the occurrence of the CMMC (Dickson et al., 2010b), it will be interesting to study the response to 5-HT of different classes of enteric neurons that are endowed with various subtypes of 5-HT receptors. 5-HT has long been identified to play an important role in GI motility and has proven to be of clinical importance (Viramontes et al., 2001; De Giorgio et al., 2007; Gershon and Liu, 2007; Grundy, 2008). Our future investigations may provide a link between Na_v_1.9 and pathological GI motor patterns. At last, we will evaluate the effect of the loss of Na_v_1.9 in enteric neurons on colonic transit.

In summary, the loss of Na_v_1.9 in enteric neurons of the mouse colon significantly affects a major pattern of motility. Our study provides the first evidence for the role of Na_v_1.9 in normal conditions. Therefore, Na_v_1.9 can no longer be regarded only as a mediator of the hyperexcitability of sensory neurons in inflammatory conditions. The model needs further study to evaluate the mechanisms underlying such changes.

### Conflict of interest statement

The authors declare that the research was conducted in the absence of any commercial or financial relationships that could be construed as a potential conflict of interest.
